# Structural basis of closed groove scrambling by a TMEM16 protein

**DOI:** 10.21203/rs.3.rs-3256633/v1

**Published:** 2023-08-18

**Authors:** Zhang Feng, Omar E. Alvarenga, Alessio Accardi

**Affiliations:** 1 Department of Anesthesiology, Weill Cornell Medical College; 2 Physiology, Biophysics and Systems Biology Graduate Program, Weill Cornell Medical College; 3 Department of Physiology and Biophysics, Weill Cornell Medical College; 4 Department of Biochemistry, Weill Cornell Medical College

## Abstract

Activation of Ca^2+^-dependent TMEM16 scramblases induces the externalization of phosphatidylserine, a key molecule in multiple signaling processes. Current models suggest that the TMEM16s scramble lipids by deforming the membrane near a hydrophilic groove, and that Ca^2+^ dependence arises from the different association of lipids with an open or closed groove. However, the molecular rearrangements involved in groove opening and of how lipids reorganize outside the closed groove remain unknown. Using cryogenic electron microscopy, we directly visualize how lipids associate at the closed groove of Ca^2+^-bound nhTMEM16 in nanodiscs. Functional experiments pinpoint the lipid-protein interaction sites critical for closed groove scrambling. Structural and functional analyses suggest groove opening entails the sequential appearance of two π-helical turns in the groove-lining TM6 helix and identify critical rearrangements. Finally, we show that the choice of scaffold protein and lipids affects the conformations of nhTMEM16 and their distribution, highlighting a key role of these factors in cryoEM structure determination.

## Introduction

Activation of phospholipid scramblases disrupts the plasma membrane asymmetry, and results in the exposure of the negatively charged lipids phosphatidylserine (PS), an important signaling molecule. Thus, lipid scrambling plays a key role in a variety of physiological processes, ranging from recognition of apoptotic cells by macrophages and blood coagulation, to viral entry, membrane fusion and repair ^1–3^. To date, three families of integral membrane proteins have been identified as lipid scramblases, the Ca^2+^-activated TMEM16s ^4–7^, the caspase-dependent XKRs ^8^, and the ATG9 proteins involved in autophagosome formation ^9–11^. Additionally, several other proteins and single-helix membrane spanning peptides have been reported to mediate lipid scrambling ^12–16^. These scramblases share broadly conserved functional characteristics, such as mediating rapid and poorly selective lipid transport ^1–3,6,7^, while displaying a remarkable structural diversity, as each family adopts a different fold ^7,9–11,17–22^. Thus, it is not known whether these diverse scramblases function according to a conserved mechanism.

**Figure 1.**
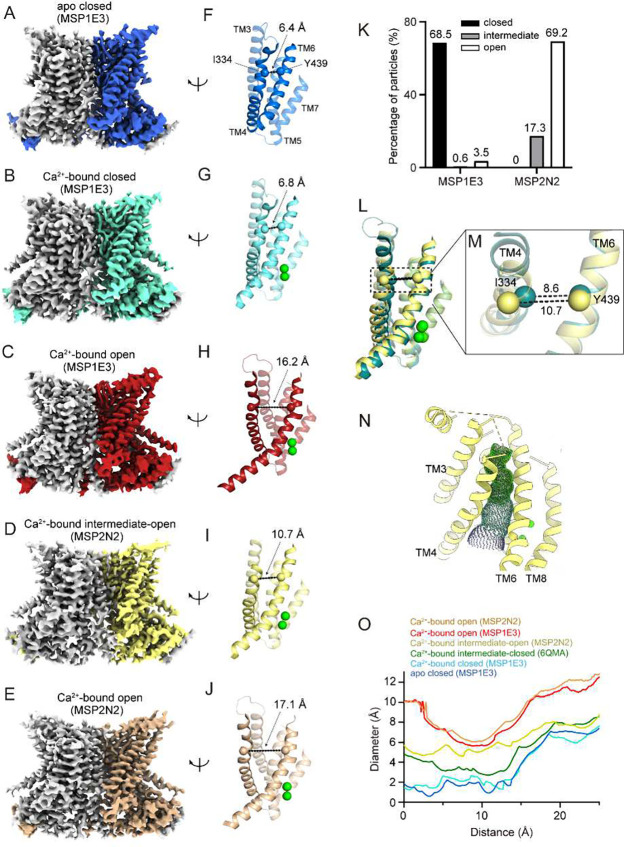
Cryo-EM structures of nhTMEM16 in the MSP1E3 and MSP2N2 nanodiscs. **A**-**E**, Cryo-EM maps of nhTMEM16 in nanodiscs formed from a 7:3 mix of DOPC:DOPG lipids and MSP1E3 (**A**-**C**) or MSP2N2 (**D**-**E**) scaffold proteins. (**A**) Ca^2+^-free, closed groove, (**B**) Ca^2+^-bound, closed groove, (**C**) Ca^2+^-bound, open groove, (**D**) Ca^2+^-bound, intermediate-open groove, and (**E**) Ca^2+^-bound, open groove. In all dimers, one protomer is colored in gray and the other in blue (**A**), cyan (**B**), red (**C**), yellow (**D**), and sand (**E**). **F-J**, The groove in the different conformations is viewed from the plane of the membrane. Transmembrane helices are shown in cartoon representations and labelled. States are colored as in (**A**-**E**). Colored spheres correspond to the position of the Cα atoms of I334 on TM4 and Y439 on TM6 and their distance is indicated. Ca^2+^ ions are shown as green spheres. **K**, The percentage of particles with a closed (black bar), intermediate (gray bar), or open (white bar) groove conformation in the datasets of Ca^2+^-bound nhTMEM16 in MSP1E3 or MSP2N2 nanodiscs. Here, intermediate does not distinguish between the intermediate-open and -closed conformations. **L-M**, Alignment of the groove helices in the intermediate-open (yellow) and previously reported intermediate-closed (PBD: 6QMA, dark green) states (**L)**, the distance between the Cα atoms of I334 on TM4 and Y439 on TM6 increases by ~2 Å in the intermediate-open **(M)**. **N**, The accessibility of the permeation pathway of nhTMEM16 in the intermediate conformation is visualized using the program Hole ^36^. **O**, The inner diameter of the permeation pathway of nhTMEM16 in MSP1E3 or MSP2N2 nanodiscs and in the published the diameter of a putative ion conduction pathway measured in different states with Hole including the Ca^2+^-bound open state in MSP2N2 (orange), Ca^2+^-bound open state in MSP1E3 (red), Ca^2+^-bound intermediate-open state in MSP2N2 (yellow), Ca^2+^-bound closed state in MSP1E3 (cyan), the previously reported intermediate-closed state in MSP2N2 (dark green) and Ca^2+^-free closed in MSP1E3 (blue).

The currently accepted paradigm for lipid scrambling, the so-called credit-card mechanism, is that scramblases provide a hydrophilic pathway that shields the polar lipid headgroups as they traverse the bilayer, while the acyl chains remain embedded in the hydrocarbon core of the membrane ^7,23^. In several dimeric TMEM16 scramblases, Ca^2+^ binding favors opening of a transmembrane hydrophilic groove which has been proposed to serve as the translocation pathway for lipid headgroups ^7,24–28^. The lipid groove is formed by TM3–7 helices of each TMEM16 protomer, and its interior is lined by hydrophilic residues ^7^ (Fig. 1A–C, F-H). In the apo conformation, the TM4 and TM6 form an extensive interface that seals the groove interior from the hydrocarbon core of the membrane (Fig. 1A, F), resulting in a closed groove. The Ca^2+^ binding sites are located between TM6–8 and their formation is enabled by a rearrangement of the intracellular portion of TM6 (Fig. 1B, G). Groove opening follows Ca^2+^ binding and entails a movement of the TM3 and TM4 helices away from TM6 ^17,18,21^ (Fig. 1C, H). However, Ca^2+^ binding does not promote groove opening in all TMEM16 scramblases as the mammalian TMEM16F exclusively adopts a Ca^2+^-bound closed groove conformation, even in conditions of maximal activity ^19,22,29^, leading to the proposal that the closed groove is also a scrambling competent state ^19,29,30^. Thus, how Ca^2+^ binding to the TM6-lined sites induces the rearrangement of TM3 and TM4 to induce groove opening in some but not all TMEM16s is not known.

Low resolution cryogenic electron microscopy (cryoEM) imaging of TMEM16 scramblases in nanodiscs showed these proteins induce a pronounced thinning of the membrane near the groove region, a distortion that is more pronounced when the groove opens ^17,21,30^. Recently, high-resolution structures of the Ca^2+^-bound fungal afTMEM16 with an open groove revealed that lipids adopt distorted poses with their headgroups positioned outside the pathway, providing the bases for membrane thinning near the open pathway ^30^. Further, functional experiments suggested that specific interactions between groove-lining residues and lipid headgroups are not required for scrambling with an open groove ^30^. Together, these observations led to the proposal that scrambling at an open groove is enabled by membrane thinning ^30^, rather than occurring via a credit card mechanism. Because membrane thinning is also observed in conditions when the groove is closed, it was proposed that TMEM16F could scramble lipids outside a closed groove ^19,30^. This proposal could also account for the basal activity of many TMEM16 scramblases in the absence of Ca^2+^ when the groove is closed ^5,7,18^. However, direct structural information on how lipids arrange near a closed groove is lacking, and it is not known whether or how their arrangement plays a role in closed groove scrambling.

One important factor to consider when interpreting cryoEM imaging experiments is the potential role of the environment in determining the conformation and state distribution of proteins. For example, the Ca^2+^-bound human TMEM16K scramblase crystalizes with an open groove but has a closed groove in single particle cryoEM imaging conditions ^18^, and the fungal nhTMEM16 adopts conformations with an open, closed, and intermediate-closed groove with roughly equal probability when reconstituted in MSP2N2 nanodiscs but is only in an open groove state when imaged in detergent micelles with cryoEM and x-ray crystallography ^7,21^. In contrast, other homologues, such as murine TMEM16F and fungal afTMEM16 are less sensitive to the environment, as they exclusively adopt a single conformation in detergent and in nanodisc environments ^17,19,22,30^. Thus, the extent to which choices of detergent, lipid and/or nanodisc scaffold protein influence the conformation of TMEM16 scramblases is poorly understood.

Here, we used cryoEM to image the fungal nhTMEM16 scramblase in lipid nanodiscs formed from different scaffold proteins and found this to affect both the conformations adopted by nhTMEM16 and their distribution. In the smaller MSP1E3 nanodiscs the scramblase preferentially adopts a closed conformation, whereas in the larger MSP2N2 nanodiscs nhTMEM16 predominantly adopts open and intermediate-open conformations. The 2.64 Å resolution structure of Ca^2+^-bound closed nhTMEM16 reveals how lipids arrange near the groove, thus showing how the scramblase thins the membrane outside a closed groove. Mutating residues that coordinate outer leaflet lipids near the closed groove impairs scrambling only in the absence of Ca^2+^, suggesting these interactions play a specific role in closed-groove scrambling. Structural analysis of the transition of nhTMEM16 from closed to open state suggests that the E313-R432 salt bridge could be involved in groove opening. Disruption of this interaction by the R432A mutation trapped nhTMEM16 in a closed-groove conformation, suggesting the salt bridge is critical for groove opening. In sum, our work provides insights into how lipids are scrambled outside a closed groove, how the groove opens, and how these processes are regulated by the lipid environment.

## Results

### The conformational landscape of nhTMEM16 depends on the environment

To understand how the environment affects the conformational landscape of TMEM16 scramblases, we focused on the fungal nhTMEM16 as this homologue can adopt multiple conformations in different reconstitution systems ^7,21^. We imaged the scramblase in the absence and presence of Ca^2+^ and in nanodiscs formed from a 7:3 mixture of 1,2-Dioleoyl-sn-glycero-3-phosphocholine (DOPC) and 1,2-Dioleoyl-sn-glycero-3-phosphatidylglycerol (DOPG) lipids. We used the MSP1E3 and/or MSP2N2 scaffold proteins that differ in their average diameter by ~3 nm ^31,32^. In 0 Ca^2+^ and in MSP1E3 nanodiscs, we obtained a single class of particles, which yielded a map with average resolution of 2.93 Å corresponding to a conformation with a closed hydrophilic groove and no density in the Ca^2+^ binding sites (Fig. 1A, F, Supplementary Fig. 1A, 2, 3). While this is similar to what was reported for nhTMEM16 in nanodiscs formed with the larger MSP2N2 and POPC:POPG lipids, there are differences in the Ca^2+^ sites and position of the cytosolic domains (see below). In contrast, the conformational distribution of nhTMEM16 changes drastically when reconstituted in 0.5 mM Ca^2+^ in MSP1E3 or MSP2N2 nanodiscs. We found that in MSP2N2 nanodiscs the majority of the particles (~69.2%) adopt an open groove conformation, ~17.3% are in an intermediate-open state (see below), and no class with a closed groove could be detected (Fig. 1D–E, I–J, Supplementary Fig. 2, 4). The remaining ~13.5% particles have poorly defined TM4 helices and could not be assigned (Supplementary Fig. 4). This is different from the findings in MSP2N2 POPC/POPG nanodiscs ^21^, where the closed groove conformation was also significantly populated. In the smaller MSP1E3 nanodiscs nhTMEM16 predominantly adopts a closed groove conformation (~68.5% of particles) (Fig. 1B, G, Supplementary Fig. 2, 5) and only a small fraction is in an open groove conformation (~3.5% of particles) (Fig. 1C, H, Supplementary Fig. 2, 5). In the remaining ~28% of particles the density around the groove region was not well resolved, suggestive of conformational heterogeneity. Classification of the symmetry-expanded and signal-subtracted protomers from this subset shows that ~16% adopt a conformation with a closed groove, ~15% are in an intermediate conformation and ~11% have an open groove (Supplementary Fig. 5–6). In the remaining ~58% protomers the density for TM4 is of insufficient quality for model building but is consistent with the groove being in closed or intermediate conformations (Supplementary Fig. 6). Mapping these protomers to the original dimeric particles allowed for a reconstruction of a nhTMEM16 dimer with one open and one closed groove (Supplementary Fig. 5), consistent with the idea that the protomers can gate autonomously. In sum, these results show that the open groove conformation of nhTMEM16 is favored >20-fold by reconstitution in the larger MSP2N2 nanodiscs (Fig. 1K).

Next, we analyzed whether the choice of nanodisc scaffold protein and lipid composition also affect the structures of the observed nhTMEM16 conformations. Unexpectedly, we found significant structural differences. In the Ca^2+^-free conformation determined in MSP1E3 DOPC/DOPG nanodiscs the Ca^2+^ binding sites are empty (Supplementary Fig. 1A), TM6 is bent, the groove is closed, and the cytosolic domains move away from each other by ~4 Å (Supplementary Fig. 1C). This rearrangement resembles that seen in afTMEM16 ^17,30^. In the previously reported putative apo nhTMEM16 structure (PBD: 6QM4; Cα r.m.s.d. 2.04 Å) ^21^ these rearrangements were not observed, likely due to the presence of an unassigned density in the Ca^2+^ binding sites in this map (Supplementary Fig. 1B). This suggests that partial ligand binding to these sites is sufficient to induce a rearrangement in the cytosolic domains. The Ca^2+^-bound open conformation determined in MSP1E3 DOPC/DOPG nanodisc is nearly identical to the previously determined open structure of nhTMEM16 determined in the larger MSP2N2 POPC/POPG discs (PDB: 6QM9) ^21^ (Cα r.m.s.d ~0.58 Å, Supplementary Fig. 1E). The open conformation in MSP2N2 DOPC/DOPG nanodiscs has a slightly more opened groove than that in POPC/POPG lipids because of a lateral displacement of the TM4 by ~1.5 Å (Cα r.m.s.d ~1.24 Å, Supplementary Fig. 1F). Finally, the intermediate conformation seen in the MSP2N2 DOPC/DOPG nanodiscs differs from the previously reported intermediate-closed one, as the TM4 is shifted further away from TM6 by ~2 Å and accompanied with a counterclockwise rotation of the extracellular portion of TM4, such that there is minimal contact between TM4 and TM6 (Cα r.m.s.d ~1.35 Å, Fig. 1L–M, Supplementary Fig. 1G). This repositioning results in a conformation where the groove, while remaining isolated from the hydrocarbon core of the membrane, forms a continuous transmembrane pore that is wide enough to accommodate water molecules and permeant ions such as K^+^ and Cl^−^ (Fig. 1N–O). This is consistent with the reported channel activity of nhTMEM16 ^33,34 35^. Therefore, we refer to this conformation as intermediate-open. Together, our results show that the scaffold protein and lipid composition affect both the distribution and structures of the conformations adopted by nhTMEM16.

### Basis of membrane thinning at the closed groove of nhTMEM16

**Figure 2.**
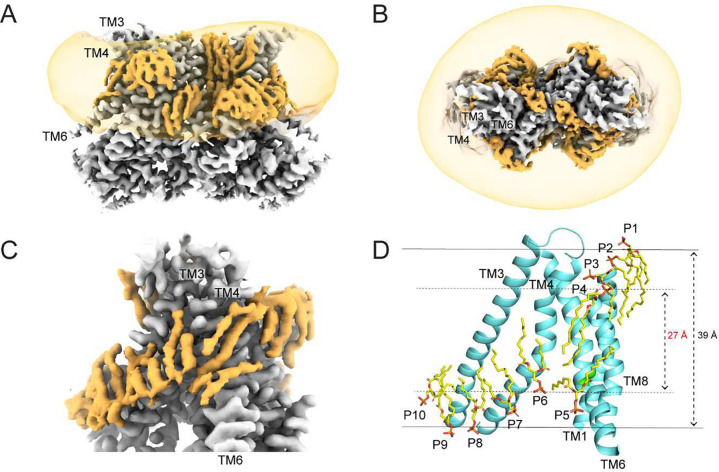
Arrangement of lipids at the closed groove of nhTMEM16. **A**-**B,** Segmented Cryo-EM map of nhTMEM16 in the Ca^2+^-bound closed state (gray) and the associated lipids (orange) viewed from the membrane plane (**A**) and from the extracellular side (**B**). The map showing the density of the nanodisc membrane is low-pass filtered to 10 Å and shown in transparent orange. **C,** View of the lipids outside of the closed groove from the plane of the membrane. **D,** Stick representation of the ten pathway lipids colored in yellow (P1-P10). Dashed arrow indicates the distance between the phosphate atoms of the last lipid from the inner (P6) and outer (P4) leaflets (~27 Å) and the measured membrane thickness (~39 Å). Lipids were built up to the phosphate atom in the head. Ca^2+^ ions are displayed as green spheres.

The cryoEM map of Ca^2+^-bound nhTMEM16 with a closed groove in MSP1E3 nanodiscs reached an average resolution of 2.64 Å, allowing us to identify 36 well-defined non-protein densities, 18 on each protomer, that could be modeled as lipids (Fig. 2A–D, Supplementary Fig. 7A-E). These densities define nearly continuous inner (IL) and outer leaflets (OL) and thus outline the protein-membrane interface (Fig. 2A–D; Supplementary Movie 1; Supplementary Fig. 7A-E). The 8 lipids near the dimer interface (D1-D8) define the inner and outer leaflets planes and adopt similar positions to those seen in Ca^2+^-bound open afTMEM16 ^30^ (Supplementary Fig. 7C-E), consistent with the idea this region undergoes minimal rearrangements during groove opening.

**Figure 3.**
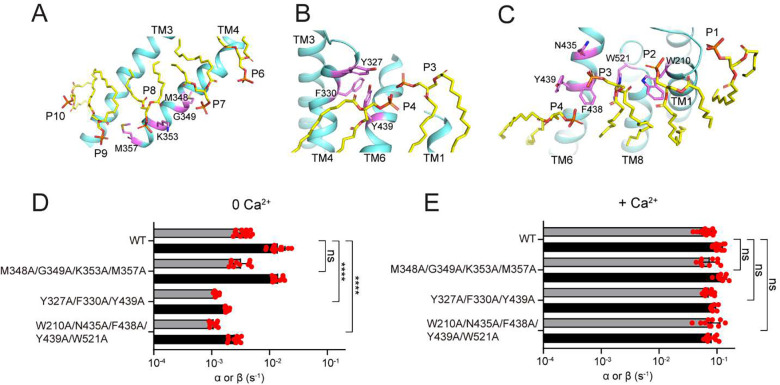
Identification of residues important for the closed groove scrambling. **A**-**C,** Residues coordinating the P7-P9 (**A**), P3-P4 (**B**) and P2-P4 lipids (**C**) in the Ca^2+^-bound closed state. **D**-**E,** Forward (α) and reverse (β) scrambling rate constants of the mutants of nhTMEM16 aimed at disrupting the protein-lipid interactions (shown in **A**-**C**) measured in 0 (**D**) or 0.5 mM Ca^2+^ (**E**). Bars are averages for α (black) and β (gray) (N ⩾ 3), error bars are S. Dev., and red circles are values from individual repeats. The statistical significance of the effects of the mutants on the scrambling rate constants was evaluated with a two-sided Student’s t-test with a Bonferroni correction. **** denotes p<10^−5^ after Bonferroni correction.

The arrangement of the 10 lipids near the pathway (P1-P10) reveals the structural basis of membrane thinning at a closed groove (Fig. 2D, Supplementary Fig. 7B). The outer leaflet P1-P4 lipids become progressively more tilted as they approach the site of TM4-TM6 contact that defines the groove closure (Fig. 2D) and their headgroups form extensive interactions with aromatic and polar residues in TM1, TM4, TM6, and TM8 (Fig. 3C). Notably, the headgroups of the P3 and P4 lipids adopt severely distorted poses (Fig. 2D) and interact with three aromatic residues, Y327 and F330 on TM4 and Y439 on TM6 (Fig. 3B). The P5 and P6 lipids delimit the inner leaflet across the wide intracellular vestibule of the pathway (Fig. 2D). The closest point of approach of the two leaflets at the closed groove is defined by the phosphate atoms of P4 and P6 which are separated by only ~27 Å, so that the hydrocarbon core of the membrane is thinned by ~30% (Fig. 2D, Supplementary Fig. 13). The Ca^2+^-bound and Ca^2+^-free closed conformations are nearly identical in this region (Supplementary Fig. 8A-E), indicating that protein-lipid interactions are likely unchanged in the two conformations. Thus, if the observed thinning is important for closed groove scrambling, then we expect these residues to play similar roles in the Ca^2+^-free and Ca^2+^-bound closed states.

### Residues coordinating outer leaflet lipids are important in closed groove scrambling

To test this hypothesis, we designed three composite mutations aimed at disrupting coordination of the OL P1-P2-P3 (W210A/N435A/F438A/Y439A/W521A) and P3-P4 lipids (Y327A/F330A/Y439A) or of the IL P6-P10 lipids (M348A/G349A/M353A/K357A) (Fig. 3A–C). In the presence of 0.5 mM Ca^2+^, when scrambling is primarily mediated by the open groove, the three constructs have WT-like rate constants (Fig. 3E). In contrast, in 0 Ca^2+^, when the groove is closed, the two constructs disrupting interactions with OL lipids display an 8- and 6-fold reduction in scrambling activity (p<10^−7^) (Fig. 3D). The scrambling rate constants of the mutant disrupting interactions with IL lipids are not statistically different from WT (P>0.4) (Fig. 3E). Thus, these mutants impair scrambling only in the absence of Ca^2+^, when the groove is closed, but have no effect in the presence of Ca^2+^, when the groove can open, suggesting that the two processes are mediated by different conformations. Therefore, we hypothesize that nhTMEM16, like afTMEM16 ^30^ and mTMEM16F ^19^, can scramble lipids with a closed groove and that remodeling of the OL lipids play a critical role in this process.

### Steps in groove opening of nhTMEM16

**Figure 4.**
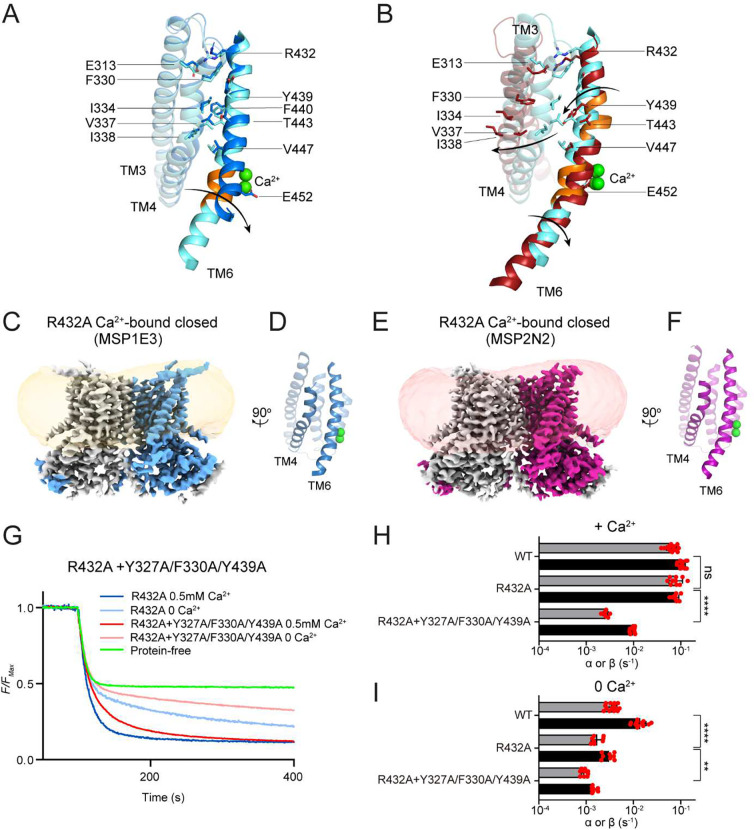
Role of the E313-R432 salt bridge in groove opening. **A**-**B**, Structural comparisons of the groove in apo (blue) vs Ca^2+^-bound closed (cyan) (**A**) and Ca^2+^-bound closed (cyan) vs open (red) states (**B**). Arrows denote rotations in the TM6. Sidechains of E313 and R432, and of the residues forming the TM4-TM6 interface are shown as sticks. π-helical turns are colored in orange. Ca^2+^ ions are displayed as green spheres. **C, E**, Cryo-EM maps of R423A nhTMEM16 in MSP1E3 (**C**) or MSP2N2 (**E**) nanodiscs. One protomer is colored in gray and the other in light blue (**C**) or magenta (**E**). The density of the nanodisc membrane is low pass filtered to 7 Å and shown in transparent orange (**C**) and red (**E**). **D, F**, Views of the groove from the plane of the membrane. TM4 and TM6 are shown in cartoon representation and labelled. Ca^2+^ ions are displayed as green spheres. **G**, Representative traces of the dithionite induced fluorescence decay in the scrambling assay for protein free liposomes (green), or R432A nhTMEM16 and the quadruple mutant R432A+Y327A/F330A/Y439A nhTMEM16 in the presence (dark blue and dark red) and absence (light blue and light red) of Ca^2+^. **H**-**I**, Forward (α) and reverse (β) scrambling rate constants of the mutants measured in 0.5 mM (**H**) or 0 Ca^2+^ (**I**). Bars are averages for α (black) and β (gray) (N ⩾ 3), error bars are S. Dev., and red circles are values from individual repeats. The statistical significance of the effects of the mutants on the scrambling rate constants was evaluated with a two-sided Student’s t-test with a Bonferroni correction. ** denotes p<0.001 and **** denotes p< 10^−5^ after Bonferroni correction.

Our structures captured the nhTMEM16 in several conformations, that likely reflect intermediates as the protein transitions from a Ca^2+^-free closed, to Ca^2+^-bound closed, intermediate, and open states (Fig. 4A–B). For simplicity, we consider only the conformations determined in MSP1E3 nanodiscs, as they have the highest resolution. In the Ca^2+^-free closed state the TM6 is in an α-helical configuration (Fig. 4A). Ca^2+^ binding induces a clockwise rotation of the intracellular portion of TM6 around A444, which results in the appearance of a π-helical turn that allows the E452 side chain to coordinate the bound Ca^2+^ ion in the both upper and lower sites (Fig. 4A, Supplementary Fig. 9A-B). When the groove opens there is a counterclockwise rotation of the central portion of TM6 (between R432 and V447, Fig. 4B), which leads to the formation of a second π-helical turn (Fig. 4B). This disrupts the tight packing of TM4-TM6 in the closed groove and thus might facilitate opening (Fig. 4B). During these rearrangements the extracellular portion of TM6 does not move much, likely because the salt bridge between R432 on TM6 and E313 on TM3 provides a stabilizing interaction (Fig. 4A–B), suggesting it might play a role in the opening transition.

### Disruption of the E313-R432 salt bridge favors a closed groove

To test this hypothesis, we used cryoEM to image R432A nhTMEM16 in MSP1E3 or MSP2N2 nanodiscs in the presence of 0.5 mM Ca^2+^ (Fig. 4C, E, Supplementary Fig. 2, 10). The maps have similar average resolutions of 3.59 Å and 3.64 Å and show that R432A nhTMEM16 adopts a single class with a fully closed groove (Fig. 4D, F). Despite multiple rounds of iterative 3D classifications on dimers and protomers using different classification parameters and software (Supplementary Fig. 10), no classes displaying an open groove could be identified. The models for Ca^2+^-bound R432A are nearly identical to that of WT Ca^2+^-bound closed nhTMEM16 in MSP1E3 nanodiscs (Fig. 2D), with Cα r.m.s.d. of ~0.93 and ~0.98 Å, and in R432A only the intracellular π-helical turn is present on TM6. The only difference from the WT structure is that the density corresponding to the extracellular portion of TM4 is weakened (Fig. 4D, F), suggesting this region becomes dynamic in the mutant. Thus, disruption of the TM6-TM3 salt bridge favors a closed groove conformation.

Next, we determined how the R432A mutation affects scrambling activity in DOPC/DOPG liposomes and found it retains WT-like activity in the presence of Ca^2+^ and a ~4-fold reduction in 0 Ca^2+^ (p<10^−5^) (Fig. 4G–I). As Ca^2+^-bound R432A nhTMEM16 only adopts a closed groove conformation, we hypothesized its activity in the presence of Ca^2+^ reflects closed-groove scrambling. We tested this hypothesis by combining the R432A mutant with the Y327A/F330A/Y439A mutant, which only impairs scrambling in 0 Ca^2+^ (Fig. 3D), when the groove is closed. We expected that if Ca^2+^ bound R432A mediates closed groove scrambling, then the quadruple mutant should display reduced activity in the presence of Ca^2+^, whereas none of the original mutants did. Results meet expectations: the R432A+Y327A/F330A/Y439A mutant displays an ~8-fold reduction in scrambling activity in 0.5 mM Ca^2+^ (p<10^−5^) (Fig. 4G–H), very similar to the effect of the triple Y327A/F330A/Y439A in 0 Ca^2+^ (Fig. 3D). In the absence of Ca^2+^ the quadruple mutant reduces scrambling activity ~9-fold (Fig. 4G, I), suggesting that the mutant does not undergo major rearrangements on Ca^2+^ binding. Together, these results suggest that R432A nhTMEM16 mediates Ca^2+^-dependent closed-groove scrambling.

### Straightening of TM6 is important for closed-groove scrambling

**Figure 5.**
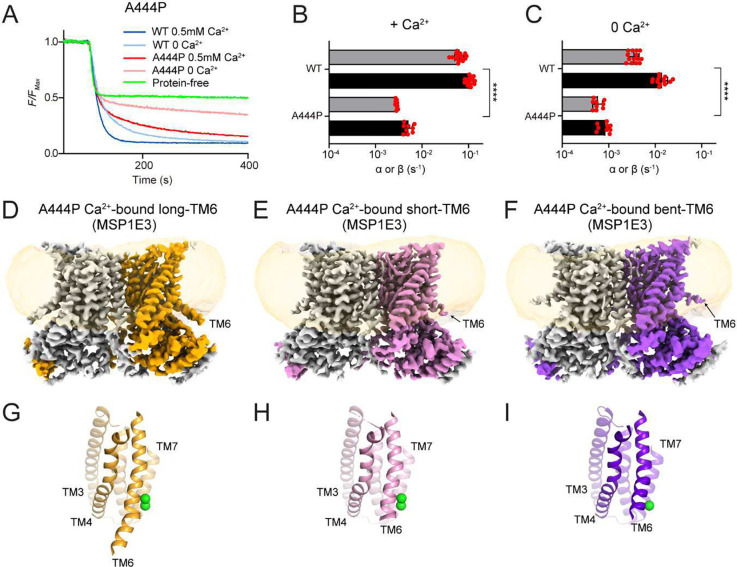
Disruption of TM6 straightening impairs lipid scrambling. **A**, Representative traces of the dithionite induced fluorescence decay in the scrambling assay in protein-free liposomes (green) or proteoliposomes reconstituted with WT and A444P nhTMEM16 in the presence (dark blue and dark red) and absence (light blue and light red) of Ca^2+^. **B**-**C**, Forward (α) and reverse (β) scrambling rate constants of the mutants measured in 0.5 mM (**B**) or 0 Ca^2+^ (**C**). Bars are averages for α (black) and β (gray) (N ⩾ 3), error bars are S. Dev., and red circles are values from individual repeats. The statistical significance of the effects of the mutants on the scrambling rate constants was evaluated with a two-sided Student’s t-test with a Bonferroni correction. **** denotes p<10^−5^ after Bonferroni correction. **D**-**F**, Cryo-EM maps of A444P nhTMEM16 in MSP1E3 nanodiscs in the long TM6 state (**D**), short TM6 state (**E**) and bent TM6 state (**F**). One protomer is colored in gray and the other in orange (**D**), pink (**E**), and purple (**F**). The density of the nanodisc membrane is low pass filtered to 7 Å and shown in transparent orange. **G**-**I**, The groove in the long TM6 (**G**), short TM6 (**H**) and bent TM6 (**I**) states is viewed from the plane of the membrane. Transmembrane helices are labelled and Ca^2+^ ions are displayed as green spheres.

One key assumption of the closed groove scrambling model is that the structural differences between the apo-closed and Ca^2+^-bound closed conformations underlie the Ca^2+^ dependence of some TMEM16 scramblases. In our structures the major difference occurring on Ca^2+^ binding is the straightening of the intracellular portion of the TM6 helix around A444 and the formation of the intracellular π-helical turn (Fig. 4A, Supplementary Fig. 9A-B). To investigate whether this rearrangement modulates closed groove scrambling we introduced a helix-bending proline at position A444. The A444P mutant displays severely impaired scrambling activity in DOPC/DOPG liposomes, with a >20-fold reduction in 0.5 mM Ca^2+^ and ~17-fold reduction in 0 Ca^2+^ (p<10^−8^) (Fig. 5A–C). Cryo-EM imaging of A444P nhTMEM16 in MSP1E3 DOPC/DOPG nanodiscs in 0.5 mM Ca^2+^ (Fig. 5D–I, Supplementary Fig. 2, 11) shows the mutant exclusively adopts conformations with a closed groove (Cα r.m.s.d. <0.4 Å to WT Ca^2+^-bound nhTMEM16 in all cases). Extensive classification results in four dimer classes: three are symmetrical and differ only in the occupancy of the Ca^2+^ binding sites and in the conformation of the intracellular portion of TM6 below 444P (Fig. 5D–F, Supplementary Fig. 12), and we refer to them as ‘long TM6’ (~1.3% of particles), ‘short TM6’ (~45% of particles), and ‘bent TM6’ (~4.5% of particles). The fourth class is asymmetric, with one protomer in the long TM6 and one in the short TM6 conformations (21.8% of particles) (Supplementary Fig. 2, 11). In the remaining ~27% of particles the density of the TM6 helices is not well defined, and therefore could not be assigned to any of the classes (Supplementary Fig. 11). Whereas in the long TM6 map the density for both Ca^2+^ binding sites is similar (Fig. 5D, G, Supplementary Fig. 12), in the short TM6 map the density in the upper Ca^2+^ binding site is weaker than that in the bottom site (Fig. 5E, H, Supplementary Fig. 12). In the bent TM6 conformation the E452 side chain on TM6 is oriented away from the Ca^2+^ binding site, and we detect weak density only for the bottom Ca^2+^ binding site (Fig. 5F, I, Supplementary Fig. 12), suggesting impaired ion binding. Thus, the A444P mutation impairs scrambling, and favors states with short or bent TM6, with weakened Ca^2+^-binding (Supplementary Fig. 12). These results suggest that the Ca^2+^-dependent straightening of TM6 might be important for closed groove scrambling.

### The effect of nhTMEM16 mutants depends on the lipid environment

**Figure 6.**
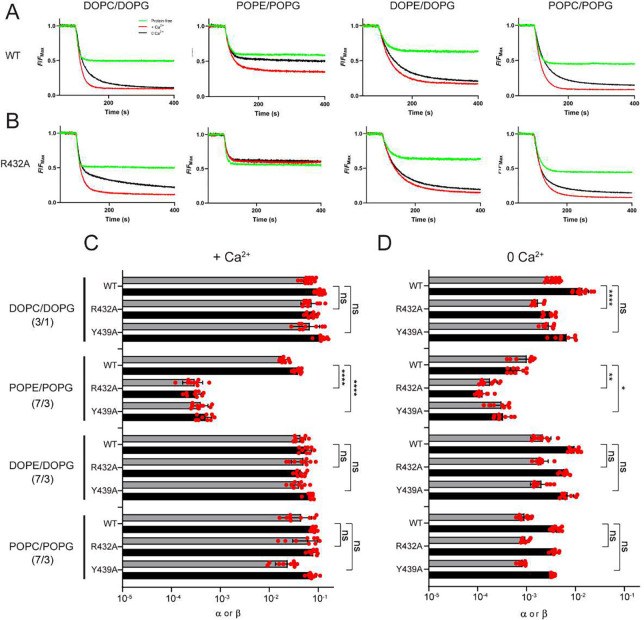
The lipid environment determines the effect of mutations of nhTMEM16. **A-B,** Representative traces of the dithionite induced fluorescence decay in the scrambling assay in protein-free liposomes (green) or proteoliposomes reconstituted with WT (**A**) or R432A (**B**) nhTMEM16 in the presence (red) and absence (black) of Ca^2+^. Liposomes were formed from a 3:1 mix of DOPC/DOPG, or a 7:3 mix of POPE/POPG, DOPE/DOPG and POPC/POPG lipids. **C**-**D,** Forward (α) and reverse (β) scrambling rate constants of WT, R423A and Y439A nhTMEM16 in the four different lipid compositions (as in **A**-**B**) in the presence of 0.5 mM (**C**) or 0 Ca^2+^ (**D**). Bars are averages for α (black) and β (gray) (N ⩾ 3), error bars are S. Dev., and red circles are values from individual repeats. The statistical significance of the effects of the mutants on the scrambling rate constants was evaluated with a two-sided Student’s t-test with a Bonferroni correction. *, **, and **** respectively denote p<5^.^10^−3^, <10^−3^, and <10^−5^ after Bonferroni correction.

Finally, we note that our results on R432A nhTMEM16 contrast with our previous report ^27^, where we showed that this and several other mutations, such as Y439A, greatly impair activity. The key difference between our current and past experiments is the membrane composition, a DOPC/DOPG mixture here and a POPE/POPG/Egg PC mixture then. To test if changes in lipid headgroups or acyl chain saturation underlie this difference we reconstituted WT and mutant nhTMEM16 in liposomes formed from DOPC/DOPG, POPE/POPG, DOPE/DOPG and POPC/POPG lipid mixtures. Consistent with previous reports ^27,34^, WT nhTMEM16 is active in all compositions, with a ~3-fold reduction in POPE/POPG membranes compared to the other conditions (Fig. 6A and 6C–D). In contrast, scrambling by R432A nhTMEM16 is reduced by >200-fold in POPE/POPG liposomes, but is WT-like in DOPC/DOPG, DOPE/DOPG and POPC/POPG membranes (Fig. 6B and 6C–D). The Y439A mutant is also only inhibited in POPE/POPG liposomes and WT-like in the others (Fig. 6C–D). Thus, scrambling activity is specifically reduced in POPE-containing membranes, as all constructs are functional in DO lipids as well as in PO membranes formed from PC/PG mixtures. This rules out specific interactions between the protein and the PE headgroup or the 16:1–18:0 acyl chains of PO lipids. While we do not have a satisfactory explanation for this observation, we note that the transition temperature (T_m_) of POPE membranes is close to room temperature, whereas those of all other tested compositions is < 0 °C ^37^. This raises the possibility that scrambling might be sensitive to the formation of local gel-like microdomains with reduced fluidity.

## Discussion

The Ca^2+^ dependent activation of TMEM16 scramblases plays a critical role in a variety of cellular signaling pathways ^1–3,6^. Despite extensive structural and functional investigations, the structural basis of their Ca^2+^-dependent activation and lipid transport mechanisms remain poorly understood. For example, TMEM16 activation is thought to entail opening of a hydrophilic groove following Ca^2+^ binding to a site formed by residues in the TM6-TM8 helices. However, it is not clear how Ca^2+^ binding facilitates the displacement of TM4 to open the groove. Further, while an open groove facilitates scrambling, the closed groove has also been proposed to be scrambling competent ^19,30^. However, the structural basis of lipid scrambling outside a closed groove remain unknown.

Our high-resolution structures of Ca^2+^-bound closed nhTMEM16 reconstituted into lipid nanodiscs allowed us to directly visualize how lipid molecules interact with the closed groove of the scramblase (Fig. 2A–D; Supplementary Movie 1; Supplementary Fig. 7A-E). We found that inner and outer leaflet lipids adopt distorted poses where their phosphate headgroups coming within ~27 Å of each other at the closed groove. This arrangement of the lipids results in a ~30% thinning of the hydrocarbon core of the membrane which will facilitate scrambling when the groove is closed. Mutants aimed at disrupting this lipid arrangement only impair scrambling by nhTMEM16 in the absence of Ca^2+^, when the groove is closed, but have no effect in the presence of Ca^2+^, when the groove can open (Fig. 3D–E). These observations suggest that the observed distortion of the lipids near the groove, and the ensuing membrane thinning, are critical for closed groove scrambling. We speculate this mechanism is likely conserved across TMEM16 scramblases for several reasons. First, mutations at similar positions also impair closed groove scrambling in afTMEM16 ^30^. Second, the membrane near the closed groove of afTMEM16 and TMEM16F is thinned to a similar extent to what we see here for nhTMEM16 ^19,21,30^ and, third, in TMEM16K two resolved detergent molecules adopt tilted poses similar to those of the P4 and P8 lipids in nhTMEM16 ^18^ (Supplementary Fig. 14A-C). These results suggest that membrane thinning is a general and evolutionary conserved mechanism for lipid scrambling by the TMEM16s. When the groove is closed, thinning is less pronounced and scrambling is slower than when the groove is open, rationalizing the Ca^2+^ dependence of this process.

Piecing together our results with past work ^7,21^ we can provide a detailed description of the molecular transitions underlying the Ca^2+^ dependent activation of nhTMEM16 (Supplementary Movie 2). To this end, we make two plausible assumptions: that the previously reported apo conformation corresponds to a partially bound state and, that the 7 observed conformations represent intermediates along the transition from apo closed to Ca^2+^ bound open. The relative stability of these conformations might be differentially affected by the membrane environment, which would result in slightly different opening trajectories. Nonetheless, we hypothesize that these states are visited -albeit briefly- during opening. Starting in the apo conformation the groove is closed, TM6 is bent, and the cytosolic domains are apart (PDBID: 8TPM) (Fig. 1A, F). Partial occupancy of the Ca^2+^ sites promotes a movement of the cytosolic domains away from the symmetry axis and a partial straightening of TM6 (PBDBID: 6QM4) (Supplementary Fig. 1C-D). Binding of both Ca^2+^ ions favors the compete straightening of TM6 and the formation of the intracellular π-helix turn, which allows the acidic side chain of E452 on TM6 to come within coordination distance of the bound ion (PDBID: 8TOI) (Fig. 1B, G, 4A, Supplementary Fig. 9A). These rearrangements weaken the TM4-TM6 packing, allowing TM4 to move and adopt the intermediate-closed (PBDID: 6QMA) and intermediate-open configurations (PDBID: 8TPN) (Fig. 1D, I), which might lead to the formation of ion conduction pores (Fig. 1N, O). The partial disengagement of the TM4 from TM6 allows the extracellular portion of TM6 between the bound Ca^2+^ ions and the R432-E313 salt bridge to rotate, resulting in the formation of a second π-helix turn at A444 (Fig. 4B). These rearrangements disrupt the TM4-TM6 interface, and likely facilitate the transition to conformations with an open groove (PBDID: 8TOL and 8TOK) (Fig. 1C, E, H, J). The role of the R432-E313 salt bridge in groove opening is supported by our finding that the R432A mutation greatly stabilizes the Ca^2+^-bound closed conformation in nhTMEM16 (Fig. 4C–F). This interaction between TM6 and TM3 is well conserved across fungal and mammalian TMEM16 homologues (Supplementary Fig. 14D-G). Interestingly, in mTMEM16F the interaction is present, but the charges are reversed, and the closed groove is the most favored conformation. Similarly, charge-reversal mutants of nhTMEM16 are non-functional in POPE-containing liposomes ^27^, suggesting that the precise arrangement of charges in the salt bridge is also important.

Our results reveal that the environment plays a critical role in determining the structures of nhTMEM16 conformations as well as the population distribution among them. Indeed, subtle changes in lipid composition, from PO to DO lipids, and/or nanodisc scaffold proteins, from MSP1E3 to MSP2N2, can affect ion binding, protein conformations, and their distributions. Specifically, in MSP1E3 DOPC/DOPG nanodiscs nhTMEM16 can adopt an apo conformation (Fig. 1A, F, Supplementary Fig. 1A) which differs from the previously reported one in MSP2N2 POPC/POPG nanodiscs because of conformational rearrangements in the cytosolic and groove regions (Supplementary Fig. 1C-D). These differences likely reflect the different occupancy of the Ca^2+^ sites, empty in our structure but partially occupied in the previous ^21^ one (Supplementary Fig. 1B). This suggests that the membrane environment might modulate TMEM16 activity by affecting ion binding, as both structures were obtained in similar Ca^2+^ buffering conditions. Structural differences are also observed in the conformations adopted in the MSP2N2 DOPC/DOPG nanodiscs compared to those seen in MSP2N2 POPC/POPG ^21^, as both the open and intermediate groove conformations in DO lipids are more open than those seen in PO lipids (Fig. 1L–M, Supplementary Fig. 1F-G). Indeed, in the intermediate-open conformation seen in DO lipids TM4 makes minimal contact with TM6 (Fig. 1N) leading to the formation of a continuous transmembrane pore with internal diameter >4.1 Å, which is sufficiently wide to be fully hydrated and to allow permeation of both chloride and potassium ions. We hypothesize this state could correspond to an ion conductive conformation of nhTMEM16. Unexpectedly, the conformations seen in the smaller MSP1E3 DOPC/DOPG nanodiscs are indistinguishable from those reported in MSP2N2 POPC/POPG, indicating that both nanodisc diameter and lipid properties contribute to stabilizing specific conformations of the protein. Strikingly, these differences in particle distributions, ligand binding state, and conformations do not correlate with changes in activity, as nhTMEM16 scrambles lipids equally well in DOPC/DOPG and POPC/POPG liposomes (Fig. 6), the Ca^2+^-free conditions are nominally identical (Fig. 1A) ^21^, and the lipid composition is the same in our MSP1E3 and MSP2N2 reconstitutions (Fig. 1K). Finally, our results suggest that reconstitution in the smaller MSP1E3 nanodiscs favors closed or intermediate conformations of nhTMEM16, as we observe a ~20-fold increase in the fraction of particles with an open groove in the larger MSP2N2 discs (Fig. 1K), where the fraction of particles with a closed groove is undetectable (Fig. 1K). This raises the possibility that unbounded membranes, such as those of liposomes, might perturb less the conformational landscape of imaged protein.

Recently, much work has been devoted to extracting free energy landscapes of proteins from the analysis of particle distributions in cryoEM imaging experiments ^38–43^. Our findings challenge the straightforward assumption that the particle distribution is a faithful reporter of the conformational landscape of the protein. The lack of correspondence between protein activity, particle distribution, and conformation confounds the correlation between the free-energy landscape of a protein, cryoEM particle counts, and activity. Rather, our results show that these distributions also reflect the contributions of the environment, and that even subtle changes in detergent, scaffold protein, or lipid constituents of nanodiscs can induce dramatic shifts in the preferred conformations adopted by the reconstituted protein. These effects should be evaluated for each protein system under consideration, as for example whereas the conformational landscape of WT nhTMEM16 is highly sensitive to the environment, that of its close homologues afTMEM16 or mTMEM16F is not ^17,19,22,30^, and even the single point mutant R432A nhTMEM16 exclusively adopts only one conformation in different nanodisc scaffolds (Fig. 4C–D). Interestingly, a recent report suggests that the conformational landscape of the pentameric ligand gated ion channel ELIC is also affected by subtle changes in the imaging environment ^44^, suggesting these considerations are generally applicable to diverse types of membrane proteins.

## Data Availability

The data that support this study are available from the corresponding author upon request. All models and associated cryoEM maps have been deposited into the Electron Microscopy Data Bank (EMDB) and the Protein Data Bank (PDB). The depositions include final maps, unsharpened maps, local refined maps, and associated FSC curves.

**Table:** 

Structure	PDB	EMDB
Apo/MSP1E3	8TPM	EMD-41477
Ca^2+^-bound open/MSP1E3	8TOL	EMD-41455
Ca^2+^-bound closed/MSP1E3	8TOI	EMD-41453
-consensus map		EMD-41457
-local refined monomer map		EMD-41458
Ca^2+^-bound open/MSP2N2	8TOK	EMD-41454
Ca^2+^-bound intermediate-open/MSP2N2	8TPN	EMD-41478
Ca^2+^-bound R432A/MSP1E3	8TPO	EMD-41479
Ca^2+^-bound R432A/MSP2N2	8TPP	EMD-41480
Ca^2+^-bound A444P long TM6/MSP1E3	8TPQ	EMD-41481
Ca^2+^-bound A444P short TM6/MSP1E3	8TPR	EMD-41482
Ca^2+^-bound A444P bent TM6/MSP1E3	8TPS	EMD-41483
Ca^2+^-bound A444P long TM6/short TM6/MSP1E3	8TPT	EMD-41484
